# A Comparison Between the Postoperative Complications of Laparoscopic Sleeve Gastrectomy (LSG) and Laparoscopic Roux-en-Y Gastric Bypass (RNYGB) in Patients With Morbid Obesity: A Meta-Analysis

**DOI:** 10.7759/cureus.31309

**Published:** 2022-11-09

**Authors:** Reda H Mithany, M Hasaan Shahid, Farukh Ahmed, Saryia Javed, Sidra Javed, Anwar Zeb Khan, Adeel Kaiser

**Affiliations:** 1 Surgery, Northampton General Hospital, Northampton, GBR; 2 Surgery, Postgraduate Medical Institute, Lahore, PAK; 3 Pediatric Surgery, Children Hospital Lahore, Lahore, PAK; 4 General Surgery, Queen Elizabeth University Hospital, Glasgow, GBR; 5 General Surgery, Lahore General Hospital, Lahore, PAK; 6 Surgery, Lahore General Hospital, Lahore, PAK

**Keywords:** morbid obesity, bariatric surgery, lsg, laparoscopic sleeve gastrectomy, rnygb, laparoscopic roux-en-y gastric bypass

## Abstract

The most successful method for treating obesity is bariatric surgery. The two most common surgeries for treating morbid obesity are the laparoscopic Roux-en-Y gastric bypass (RNYGB) and the laparoscopic sleeve gastrectomy (LSG). However, there has not been a thorough analysis of the differences in their adverse effects. The aim of this study was to analyze if RNYGB and LSG had comparable postoperative complications and mortality. To that end, results from trials comparing those who underwent RNYGB and those who underwent LSG were combined. We explored the Cochrane Library, PubMed, EMBASE, and Web of Science databases for collecting pertinent data, and 10 RCTs were included in the study. Standard deviations were used to determine the risk ratio (RR) and the 95% confidence interval (CI). No substantial difference in mortality was observed between the two procedures. However, our pooled analysis showed that patients who underwent RNYGB needed some reoperation at a higher rate compared to those who had LSG, with a pooled RR of 0.64 (95% CI: 0.42-0.98; p=0.04). Patients who had LSG suffered from fewer postoperative sequelae. While the risk of other complications was higher in RNYGB, our analysis showed that the frequency of gastroesophageal reflux disease (GERD) after LSG was greater than after RNYGB, with a pooled RR of 4.00 (95% CI: 2.55-6.28; p<0.001). Based on the above-mentioned findings, RNYGB and LSG had comparable mortality rates; however, patients who underwent LSG had a reduced risk of complications and reoperations after surgery compared to those who had RNYGB.

## Introduction and background

Bariatric surgery is considered the most successful method for treating obesity. Long-term, stable weight loss of more than 50% is associated with a reduced risk of mortality and cardiac problems, improved health, and enhanced quality of life. These outcomes have been attributed to anticipated changes in cell function, tissue-specific insulin sensitivity, bile acid concentration, and the gut microbiota, in addition to calorie restriction and altered connections between the gastrointestinal system and the brain [1].

To address morbid obesity, well over 50 different surgical procedures have been proposed and attempted until now. Hence, one may conclude that the best or most suited method of operation has yet to be devised. Moreover, this fact also highlights the passion and creativity of those who advocate for bariatric surgery. Historically, six major surgical procedures have been effective in inducing principal weight reduction and have had considerable influence on the field. These operations are as follows, in the chronological order of when they were first employed by the medical community: jejunoileal bypass (JIB), laparoscopic Roux-en-Y gastric bypass (RNYGB), vertical banded gastroplasty (VBG), biliopancreatic diversion (BPD), or duodenal switch (DS), adjustable gastric banding (AGB), and laparoscopic sleeve gastrectomy (LSG). In addition, it is vital to highlight the following three technological advancements in this field: gastric stimulation, vagal blocking, and, most critically, banded RNYGB [2].

RNYGB and LSG are the most common and standard surgical procedures for treating obesity. During RNYGB, the stomach is surgically reshaped to create a small pocket distant from the gastroesophageal junction. It bypasses the stomach, duodenum, and the first part of the jejunum since it attaches directly to the small intestine. In contrast, SG results in the formation of a narrow gastric tube following the stomach's lesser curvature [1].

Several meta-analyses demonstrating the effectiveness of both these routinely conducted techniques have been reported. However, to our knowledge, no study has directly compared the potential postoperative outcomes of these two procedures.

## Review

Materials and methods

Data Collection

We conducted a search on the databases PubMed, EMBASE, the Web of Science, and the Cochrane Library from inception until April 5, 2022. We specifically looked for the following terms: bariatric surgery, sleeve gastrectomy, LSG, SG, gastric bypass, RYGB, RNYGB, and obesity. The bibliographies of selected studies were also combed through. Study titles and abstracts were read by two separate reviewers. After finding supporting evidence, the whole text was obtained for further examination. The inclusion and exclusion criteria are presented in Table 1. 

**Table 1:** Study inclusion and exclusion criteria

Parameters	Inclusion criteria	Exclusion criteria
Study design	Randomized controlled trial	Uncontrolled or non-randomized studies
	English publication	Conference proceedings, abstracts; letters; editorials; expert opinions; reviews; or case reports
	Humans	Animals
Participants and intervention	Patients underwent laparoscopic sleeve gastrectomy	Patients underwent other bariatric procedures or re-operations
	Patients underwent laparoscopic Roux-en-Y gastric bypass	
Comparison	Morbidity and mortality	Weight loss and positive outcomes

Quality Assessment

This meta-analysis was conducted by two trained assessors who independently rated the quality of all of the papers included [3-12]. All RCTs were assessed for bias, and graphs were created to show the results.

Data Extraction

Two reviewers independently collected data to compare the adverse effects of laparoscopic RNYGB and LSG, and their disagreements were addressed by reaching a consensus via a discussion. A consistent form was used to compile the research data, including publication year, place of origin, study design, and primary outcomes. The data were entered into RevMan 5.4 software for further investigation.

Statistical Analysis

Results from trials comparing those who underwent RNYGB and those who underwent LSG were combined. Standard deviations were used to determine the risk ratio (RR) and the 95% confidence interval (CI). The heterogeneity of the studies was evaluated by using the chi-squared Q statistic test, and the results were quantified using the p-value and the I^2^ statistic, which may take on values between 0 and 100%. The pooled RRs were determined by utilizing a random-effects model and a significance threshold of p≤0.10. When statistical heterogeneity was absent (p>0.010), the Mantel-Haenszel approach was used as part of a fixed-effects model. When the 95% CIs for the pooled standardized mean differences (SMD) and the pooled RR did not overlap, the results were considered statistically significant. This study has adhered to the Preferred Reporting Items for Systematic Review and Meta-Analyses (PRISMA) guidelines.

Results

Included Studies, Study Characteristics, and Quality Assessment

**Figure 1:**
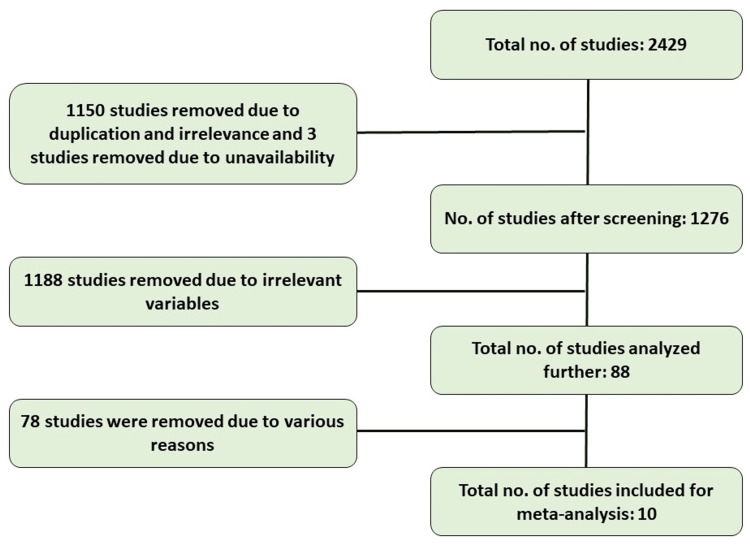
PRISMA flow diagram depicting the selection of articles for review PRISMA: Preferred Reporting Items for Systematic Reviews and Meta-Analyses

After removing duplicates, the titles and abstracts of 1276 papers were evaluated. Complete texts of the remaining 88 studies were retained for additional analysis after 1188 papers were eliminated. Following a thorough evaluation of all full-text articles, 78 were deemed ineligible for various reasons, leaving only 10 randomized controlled trials for the final analysis [3-12]. These studies originally involved 1222 people, but with 110 persons being lost to follow-up, we were left with a patient population of 1112 participants for this meta-analysis (Figure 1). Sample sizes varied from 29 to 248 patients in the trials.

**Figure 2:**
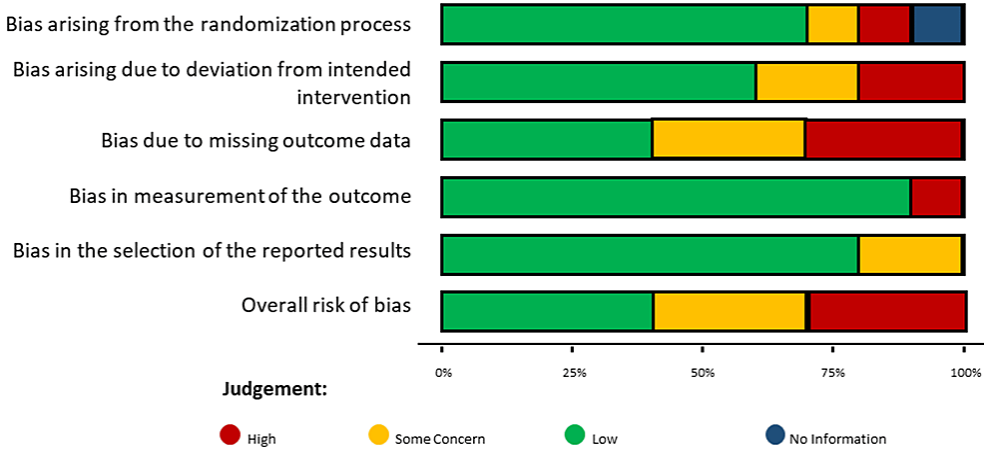
Assessment of the overall risk of bias

**Figure 3:**
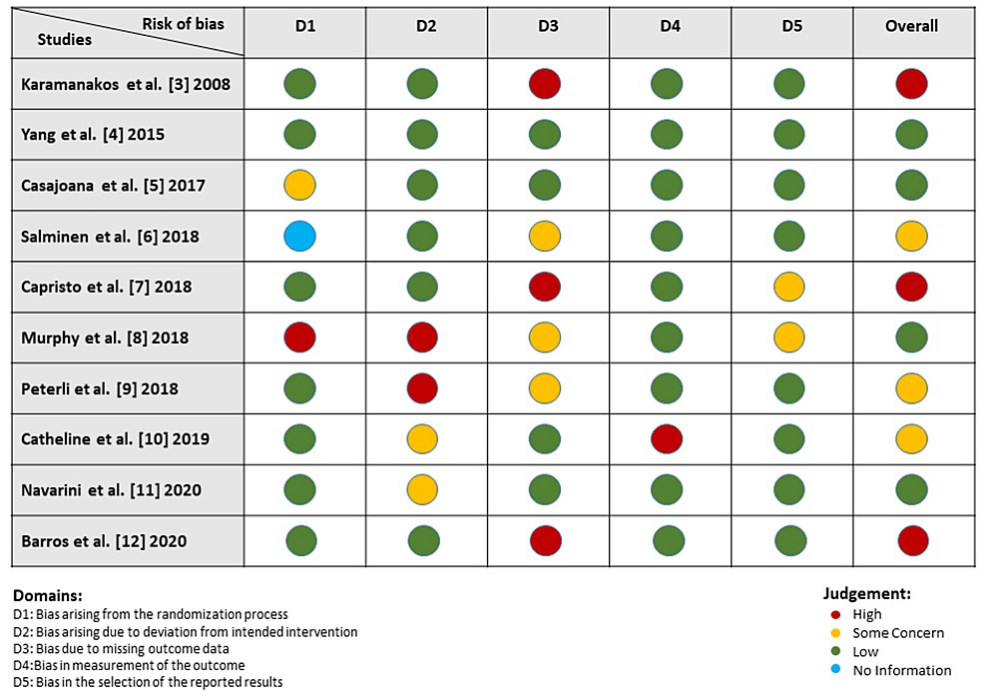
Risk of bias summary

Graphs showing the risk of bias were created. Each RCT's risk of bias is presented as a percentage of the total risk across all trials in Figure 2, while Figure 3 shows the risk of specific forms of bias. The risk of bias graphs for the RCTs demonstrated that the methodological quality was often high, particularly regarding selection and reporting biases. Twelve months of morbidity and mortality were evaluated and compared among all the studies.

Comparison of the Incidence of Intra-abdominal Hemorrhage and Abscess Formation Between LSG and RNYGB

**Figure 4:**
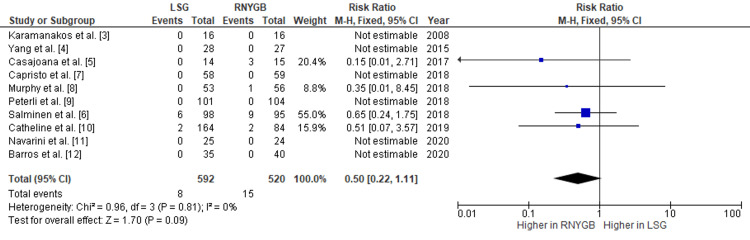
Comparison of hemorrhage incidence between RNYGB and LSG LSG: laparoscopic sleeve gastrectomy; RNYGB: Roux-en-Y gastric bypass

**Figure 5:**
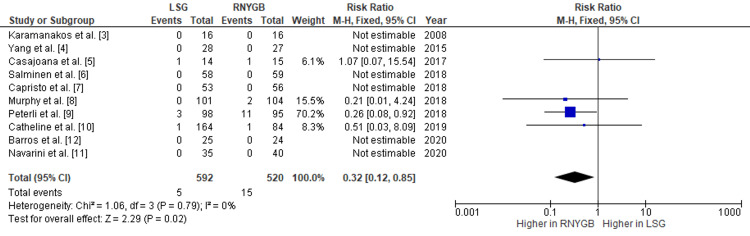
Comparison of intra-abdominal abscess incidence between RNYGB and LSG LSG: laparoscopic sleeve gastrectomy; RNYGB: Roux-en-Y gastric bypass

The pooled analysis had shown that RNYGB was associated with a higher incidence of hemorrhage, with a pooled RR of 0.50 (95% CI: 0.22-1.11; p=0.09) (Figure 4), and analysis also showed that RNYGB had more incidences of intra-abdominal abscess formation after the operation, with a pooled RR of 0.32 (95% CI: 0.12-0.85; p=0.02) (Figure 5). Since there was no substantial heterogeneity between the trials, the data was analyzed using a fixed-effects model.

Comparison of the Incidence of Internal Hernia Formation and Gastroenteric Leak Between LSG and RNYGB

**Figure 6:**
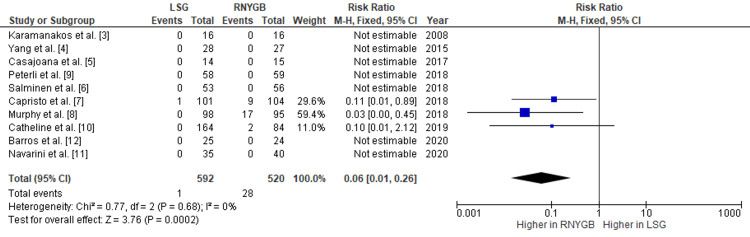
Comparison of internal hernia formation between RNYGB and LSG LSG: laparoscopic sleeve gastrectomy; RNYGB: Roux-en-Y gastric bypass

**Figure 7:**
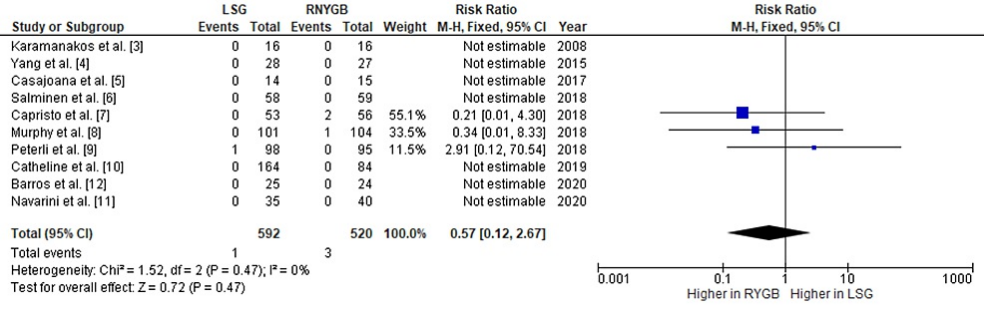
Comparison of gastroenteric leak incidence between RNYGB and LSG LSG: laparoscopic sleeve gastrectomy; RNYGB: Roux-en-Y gastric bypass

Our pooled analysis showed that RNYGB had a higher incidence of internal hernia formation as compared to LSG, with a pooled RR of 0.06 (95% CI: 0.01-0.26; p=0.002) (Figure 6). Also, the risk of gastric/enteric leak was also observed more in RNYGB, with a pooled RR of 0.57 (95% CI: 0.12-2.67; p=0.47) (Figure 7). Given the lack of significant heterogeneity between the trials, the data were analyzed using a fixed-effects model.

Comparison of the Incidence of Gastroesophageal Reflux Disease (GERD) and Gastric Ulceration Between LSG and RNYGB

**Figure 8:**
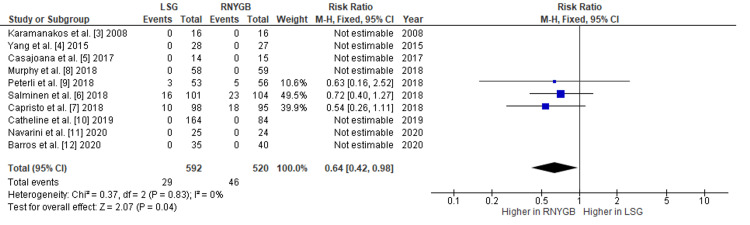
Comparison of GERD incidence between RNYGB and LSG GERD: gastroesophageal reflux disease; LSG: laparoscopic sleeve gastrectomy; RNYGB: Roux-en-Y gastric bypass

**Figure 9:**
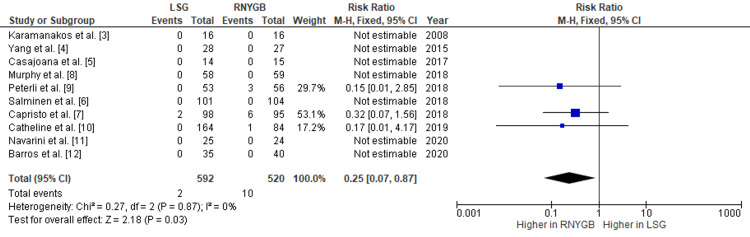
Comparison of gastric ulceration incidence between RNYGB and LSG LSG: laparoscopic sleeve gastrectomy; RNYGB: Roux-en-Y gastric bypass

Our pooled analysis showed that the frequency of GERD after LSG was greater than that in RNYGB, with a pooled RR of 4.00 (95% CI: 2.55-6.28; p<0.001) (Figure 8). Postoperatively, the risk of gastric ulceration was observed more in RNYGB, with a pooled RR of 0.25 (95% CI: 0.07-0.87; p=0.03) (Figure 9). Given the lack of significant heterogeneity between the trials, the data were analyzed using a fixed-effects model.

Comparison of the Incidence of Reoperation and Other Complications Between LSG and RNYGB

**Figure 10:**
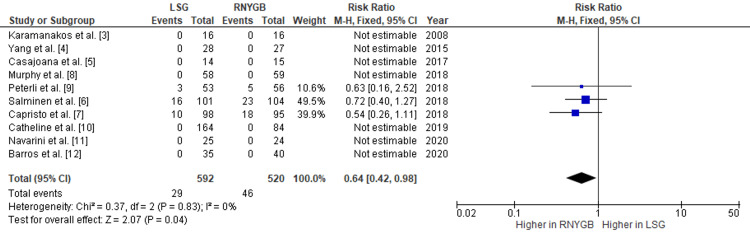
Analysis of RNYGB and LSG reoperation rates LSG: laparoscopic sleeve gastrectomy; RNYGB: Roux-en-Y gastric bypass

**Figure 11:**
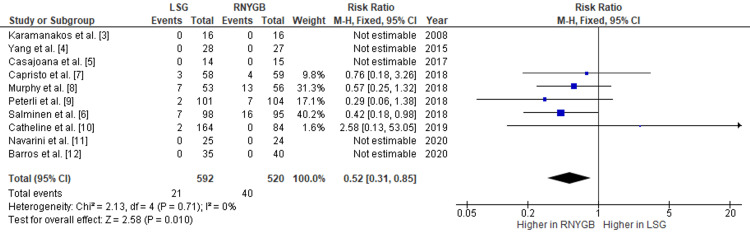
Comparison of other complications between RNYGB and LSG LSG: laparoscopic sleeve gastrectomy; RNYGB: Roux-en-Y gastric bypass

Our pooled analysis showed that patients who underwent RNYGB needed some reoperation at a higher rate as compared to those who had LSG, with a pooled RR of 0.64 (95% CI: 0.42-0.98; p=0.04) (Figure 10). Also, the risk of other complications was observed more in RNYGB, with a pooled RR of 0.52 (95% CI: 0.31-0.85; p=0.01) (Figure 11). Given the lack of significant heterogeneity between the trials, the data were analyzed using a fixed-effects model. Other complications observed were gastric outlet obstruction, fistula formation, dehydration, superficial wound infection, pneumonia, and stricture formation. 

Comparison of the Incidence of Mortality Between LSG and RNYGB

**Figure 12:**
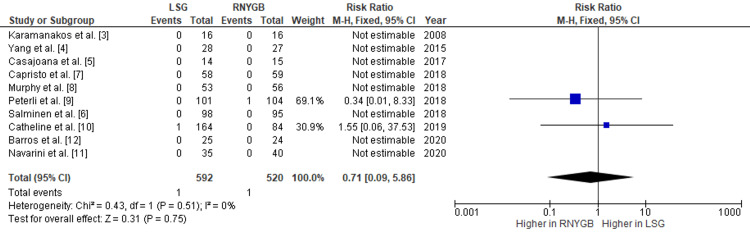
Comparison of mortality rates between RNYGB and LSG LSG: laparoscopic sleeve gastrectomy; RNYGB: Roux-en-Y gastric bypass

There were no substantial differences in postoperative patient mortality between the two procedures. Out of 1112 patients, two expired, one in each group, and were mentioned because of postoperative complications, with a pooled RR of 0.71 (95% CI: 0.09-5.86; p=0.75) (Figure 12).

Discussion

Bariatric surgery offers several advantages, including weight reduction and resolution of type II diabetes as well as other obesity-related complications [13]. Weight reduction is associated with improving and avoiding metabolic and cardiovascular disorders in the short term. However, it is uncertain if there are any detrimental consequences associated with these treatments that should be addressed.

Despite the difficulties and likelihood of reoperations associated with RNYGB, all studies indicate that the incidence of GERD following LSG is more significant than that after RNYGB, with a pooled RR of 3.13 (95% CI: 2.01-4.87; p<0.001). Gu et al. also found comparable results and indicated that obese individuals undergoing RNYGB had a decreased risk of new-onset or worsening GERD than those undergoing LSG [14].

RNYGB and LSG are the most commonly performed bariatric operations. Their adverse effects and mortality rates have not been compared. This analysis compared with LSG in terms of mortality and negative effects. Our results revealed that RNYGB and LSG were comparable in the context of mortality. However, LSG was associated with less reoperation and postoperative morbidity rates. In addition, obstruction, intraperitoneal abscess development, pleural empyema, occlusion of the biliopancreatic limb, and leaking at the gastrojejunostomy were documented as consequences of sleeve gastrectomy by Zhao et al. [13].

Our pooled analysis indicated that RNYGB patients needed reoperation at a higher rate than LSG patients, with a pooled RR of 0.64. In contrast, Chaar et al. observed that the incidence of at least one procedure or reoperation after RYGB was significantly higher when compared to SG (2.8% vs. 2.5%) [15].

Our study revealed that RNYGB had a pooled RR of 0.64 (95% CI: 0.42-0.98; p=0.04) for postoperative gastric ulcers. Zak et al. reported similar results: RNYGB was associated with more nonhealing ulcers than LSG [16]. In their study, Poublon et al. found that RNYGB has a higher rate of morbidity due to ulceration, internal herniation, and biliary reflux necessitating reoperation [17].

Furthermore, Grönroos et al. stated that the cumulative morbidity rate was 24% for sleeve gastrectomy and 28.6% for RNYGB (p=0.42). The risk of late mild complications between five and seven years was 5% for LSG, while it was 3.4% for RNYGB (p=0.75). The rate of late significant complications was 0.8% for LSG and 2.5% for RNYGB (p=0.37). These major morbidities were reoperations [18]. Our findings are also comparable to their outcomes.

## Conclusions

This meta-analysis revealed that RNYGB and LSG are comparable in terms of mortality rates. In contrast, patients who underwent LSG had fewer complications after surgery and a lower risk of reoperation compared to those who received RNYGB. However, LSG may exacerbate GERD symptoms and potentially initiate GERD. Based on our overall findings, LSG should be chosen over RNYGB for bariatric surgery. However, this should be decided only after taking the patient's other medical conditions into account.
